# First isolation of nontoxigenic *tox* gene-bearing *Corynebacterium ulcerans* from a diphtheria case in Minas Gerais, Brazil

**DOI:** 10.1007/s42770-026-01878-z

**Published:** 2026-03-02

**Authors:** Amanda Couto Calazans Silva, Lincoln de Oliveira Sant’Anna, Priscila Cristina Nunes Soares, Tayná do Carmo Sant’Anna Cardoso, Juliana Nunes Ramos, Ana Luíza Mattos-Guaraldi, Louisy Sanches dos Santos

**Affiliations:** 1 Laboratory of Diphtheria and Corynebacteria of Clinical Relevance, Department of Microbiology, Immunology and Parasitology, Rio de Janeiro State University, Avenida Vinte e Oito de Setembro, 87 - Fundos, 3º andar. Vila Isabel, Rio de Janeiro, RJ CEP.: 20 551-030 Brasil; 2 Laboratory of Microbiological Control, Institute of Technology in Immunobiologicals, Oswaldo Cruz Foundation, Rio de Janeiro, RJ Brasil

**Keywords:** Diphtheria, *Corynebacterium ulcerans*, Diphtheria toxin, NTTB

## Abstract

**Introduction:**

Diphtheria is a severe, vaccine-preventable infection caused mainly by *Corynebacterium diphtheriae*, but other species within the *C. diphtheriae* complex, including *C. ulcerans*, can also carry the diphtheria toxin (DT) coding gene (*tox*). Strains classified as “nontoxigenic toxin-gene bearing” (NTTB) harbor the *tox* gene but do not produce functional DT, although reactivation remains possible. This study reports the first isolation of a NTTB *C. ulcerans* strain from a diphtheria case in Brazil.

**Methods:**

A bacterial strain was isolated in Minas Gerais (2019) and subjected to phenotypic, molecular, and phylogenetic analyses. Identification was performed using MALDI-TOF MS, multiplex PCR (mPCR), and sequencing of the 16S rRNA and *rpo*B genes, followed by phylogenetic inference. Toxigenicity was assessed by mPCR and the modified Elek test. Antimicrobial susceptibility was determined by the disk diffusion method according BrCAST guidelines.

**Results:**

MALDI-TOF identified the isolate as *C. ulcerans*. The identification was confirmed by phylogenetic analysis of the *rpo*B gene. The strain carried the *tox* gene, but the Elek test did not show the production of DT, classifying the isolate as NTTB. Antimicrobial testing showed susceptibility to most drugs, with the susceptible, increased exposure profile to benzylpenicillin and ciprofloxacin. No resistance was detected.

**Conclusion:**

Although non-toxigenic, the isolation of a *tox*-positive *C. ulcerans* strain highlights the potential risk for re-emergence of diphtheria in Brazil. Continuous genomic surveillance, investigation of zoonotic reservoirs, and maintenance of high vaccination coverage remain crucial to prevent future outbreaks.

## Introduction

Diphtheria is a vaccine-preventable acute infectious disease that mainly affects the respiratory tract and occasionally the skin. It is potentially fatal due to the action of diphtheria toxin (DT), a potent exotoxin that has a tropism for the myocardium, central nervous system, kidneys, and adrenals [1, 2]. In addition to *Corynebacterium diphtheriae*, which is the main etiologic agent of classical diphtheria, *C. ulcerans*, *C. pseudotuberculosis*, *C. belfantii*, *C. rouxii*, *C. silvaticum*, and *C. ramonii* are considered potentially DT-producing species and currently form the *C. diphtheriae* complex [3–6]. However, to the best of our knowledge, toxigenic isolates of *C. belfantii* have not yet been reported.

The production of DT relies on the lysogenization of the microorganism by corynebacteriophages carrying the *tox* gene. However, “nontoxigenic tox gene-bearing” (NTTB) strains, which are *tox*-positive but do not express the DT due to deletions in the *tox* gene, have been isolated worldwide. Because toxin production can potentially be reactivated, the circulation of these strains represents a possible risk for the re-emergence of diphtheria [7].

In the present study, we report, for the first time, the isolation of a NTTB *C. ulcerans* strain from a diphtheria case in Brazil.

## Materials and methods

### Origin and preliminary identification of the bacterial isolate

In October 2019, a 32-year-old female patient, residing in a municipality of the metropolitan region of Belo Horizonte, Minas Gerais, Brazil, sought medical care presenting with prostration, cervical edema, fever, and a tonsillar pseudomembrane. The patient had no history of diphtheria vaccination and reported no contact with suspected or confirmed cases. Nasopharyngeal and oropharyngeal swabs were collected for laboratory investigation of diphtheria. The samples were forwarded, through the epidemiological surveillance system of the State of Minas Gerais, to the Laboratory of Diphtheria and Corynebacteria of Clinical Relevance at Rio de Janeiro State University, a collaborating center of the Brazilian Ministry of Health, for molecular identification and toxigenicity testing.

The swabs were seeded in 5% sheep’s blood agar (PlastLabor, Brazil) and incubated at 37 °C for 48 h. Morphological evaluation of the grown colonies, as well as the Gram staining, was performed. The catalase, CAMP (Christie, Atkins, and Munch-Petersen), and the DNase tests were also performed for the initial identification screening of the suspected colonies isolated from the oropharyngeal swab. The isolate was preserved in Trypticase Soy Broth with 15% glycerol (PlastLabor) at −20 °C and − 80 °C.

Preliminary molecular identification of the isolate was carried out using Matrix-Assisted Laser Desorption Ionization-Time of Flight mass spectrometry - MALDI-TOF MS in the equipment Microflex (Bruker Daltonics, France) by the method of direct identification through the bacterial colony, as previously described by Theel and collaborators [8], using the Biotyper system’s automation control and the current Bruker Biotyper 3.1 software and library. For the analysis, the isolate was previously cultivated in Trypticase Soy Agar (HiMedia, India) at 37°C for 48 h.

### Antimicrobial susceptibility testing

The susceptibility to antimicrobial agents was determined by the disk diffusion method according to the Brazilian Committee for Antimicrobial Susceptibility Testing (BrCAST) [9], using the *C. diphtheriae* and *C. ulcerans* table for interpretation of the results [10].

The following antimicrobials were tested: benzylpenicillin (1 U), cefotaxime (5 µg), meropenem (10 µg), ciprofloxacin (5 µg), erythromycin (15 µg), tetracycline (30 µg), linezolid (10 µg), rifampicin (5 µg), and trimethoprim-sulfamethoxazole (23.75 µg–1.25 µg). Results were obtained after incubation in a 5% CO_2_ atmosphere at 35 ± 1 °C for 40–44 h. The quality control of the tests was also carried out as recommended by the BrCAST document using the *Streptococcus pneumoniae* ATCC 49619 strain.

### DNA extraction and purification

Genomic DNA extraction was carried out using the QIAamp DNA Blood Mini Kit (QIAGEN, Germany) followed by adaptations carried out in the laboratory and by Nakao & Popovic [11] to obtain a better DNA yield. The modification consisted of adding 2 µl of mutanolysin (5000 U/ml; Sigma Aldrich, USA) to the lysis solution recommended by the kit and reducing the final DNA elution volume by using 100 µl ultrapure water (Sigma Aldrich).

### Molecular identification and determination of toxigenic potential

To determine the toxigenicity potential of the isolate and confirm the species identification provided by the MALDI-TOF, a multiplex PCR assay was performed according to the protocol described by Torres and collaborators [12], using oligonucleotides targeting the genes *rpo*B, *dtx*R, 16S rRNA, and *pld* for the molecular differentiation of the species *C. diphtheriae*, *C. ulcerans*, and *C. pseudotuberculosis*, and the *tox* gene for presumptive assessment of toxigenicity. As controls for the species, were used the strains *C. diphtheriae* ATCC 27010^T^ (*tox*-), *C. diphtheriae* ATCC 27012 (*tox*+), *C. ulcerans* 809, and *C. pseudotuberculosis* 1002. Strains ATCC 27010 and ATCC 27012 are also used to control toxigenic potential.

### Modified Elek test

A modified Elek test was used to confirm DT production as recommended by the World Health Organization [13] and described by Engler and collaborators [14]. To carry out the test, the control strains *C. diphtheriae* ATCC 27010^T^ (*tox*-; non-toxigenic) and *C. diphtheriae* ATCC 27012 (*tox*+; toxigenic) were used.

### Sequencing of the 16 S rRNA and RpoB genes

The sequencing of 16S rRNA and *rpo*B genes was performed for definitive species identification. The oligonucleotides PA F (AGA GTT TGA TCC TGG CTC AG) and 1492 R (TAC GGY TAC CTT GTT ACG ACT T) were used to amplify the 16S rRNA [15, 16]. The amplification and sequencing of the polymorphic region of the *rpo*B gene were performed using the oligonucleotides C2700 F (CGW ATG AAC ATY GGB CAG GT) and C3130 R (TCC ATY TCR CCR AAR CGC TG) as described by Khamis and collaborators [17]. Both amplified PCR products were purified with the QIAquick PCR Purification Kit (QIAGEN) according to the manufacturer’s instructions. Sequencing reactions were performed using the BigDye Terminator v3.1 Cycle Sequencing Kit (Applied Biosystems, Thermo Fisher Scientific, USA) following standard protocols. Sequencing was performed on the Applied Biosystems 3500 Genetic Analyzer (ABI3500) platform (Applied Biosystems, Thermo Fisher Scientific). The oligonucleotides PA F, 926 F (AAA CTY AAA KGA ATT GAC GG) [18], 519 R (GWA TTA CCG CGG CKG CTG) [19], 1093R (GTT GCG CTC GTT GCG GGA CT) [20], and 1492R [16] were used for sequencing the 16S RNA gene. The raw files were analyzed using the Geneious Prime software version 2024.0.7. Low-quality reads were trimmed, and the consensus strand of the 16S rRNA gene was then generated. The same procedure was performed with the *rpo*B gene sequencing reads. 

16S rRNA gene sequence was compared with type strain sequences available on the EzBioCloud Server (https://www.ezbiocloud.net) [21]. The *rpo*B gene sequence was compared with type strain sequences using the BLAST program [22] available on the National Center for Biotechnology Information website. (https://www.ncbi.nlm.nih.gov) [23].

### Phylogenetic analysis

For phylogenetic analysis, the sequences most closely related to the 16S rRNA gene of the isolate were used. To perform the phylogenetic study of the *rpo*B gene, the sequences of the most closely related species indicated by the analysis of the gene sequence of the 16S rRNA gene were used.

The sequences were aligned using Mafft software version 7 [24] through the PhyloSuite program version 1.2.3 [25]. Subsequently, the aligned sequences were submitted to ModelFinder program [26] for analysis and choice of the appropriate substitution model.

Finally, Bayesian phylogenetic inference was conducted using MrBayes [27], integrated into PhyloSuite, under HKY + F model (16 S rRNA) and SYM + I model (*rpo*B). Both analysis was performed with two independent runs, each with four Markov chains (one cold and three heated), run for 20.000.000 generations with sampling every 100 generations. The initial 25% of generations were discarded as burn-in.

The consensus phylogenetic trees were generated automatically, and posterior probability values were used as node support. Tree visualization and editing were performed using FigTree version 1.4.4 [28].

### Sequence deposit

The 16S rRNA and *rpo*B gene sequences have been deposited in the GenBank public repository under accession numbers PV882361 and PV892727, respectively.

### Ethical approval

This study was approved by the Research Ethics Committee of Hospital Universitário Pedro Ernesto (CEP/HUPE– CAAE: 90154324.8.0000.5259; approval nº. 7.755.182).

## Results and discussion

Although the number of diphtheria cases have been in decline since implementation of effective immunization programs with the diphtheria toxoid, diphtheria remains endemic in many countries and outbreaks of the disease have been reported in some regions, including European countries [29]. In Brazil, 55 diphtheria cases have been confirmed in the past 10 years. The highest number was recorded in 2015 (16 cases), most of them in Pernambuco (11 cases) [30]. In the present study, the bacterial isolate recovered from a diphtheria case that occurred in Minas Gerais, a state where only 3 cases were reported in the last decade.

The bacterial strain presented non-hemolytic, medium (1 mm), and round colonies with well-defined edges and a light beige color. The catalase test was positive, while the DNase was weakly positive. The isolate was negative for the CAMP factor. MALDI-TOF identified the isolate as *C. ulcerans*, presenting a score of 2,200, which indicates a reliable identification according to the manufacturer. In the present study, the strain was designated as *C. ulcerans* 2965.

For decades, identifying corynebacteria has been a challenge regardless of the technology used [31]. Although MALDI-TOF MS for the identification of microorganisms is an important tool for clinical laboratories, helping to identify rare and relevant microorganisms, the difficulty in identifying *Corynebacterium* spp. still exists [32]. Suwantarat and collaborators [33], in their study comparing conventional biochemical identifications with MALDI-TOF, observed 99.5% correct identification at the genus level; however, they obtained 88.7% reliable identification at the species level. Thus, in the present study, an additional molecular method was applied to confirm the species identification of the isolate.

Molecular identification using the mPCR technique yielded results compatible with the strain *C. ulcerans* 809 used as a species-specific positive control for the analysis, amplifying in the isolate’s template DNA the fragments generated by the pairs of oligonucleotides for the 16S rRNA and *rpo*B genes. However, due to the recent taxonomic descriptions of the new species belonging to the *C. diphtheriae* complex, *C. belfantii* [3], *C. rouxii* [4], *C. silvaticum* [5], and *C. ramonii* [6], and to the close relationship between these species, the sequencing of the 16S rRNA and *rpo*B genes was carried out to reliably achieve species-level identification.

Gene similarity analysis carried out by local alignment, the isolate obtained values ​​compatible with the species *C. ulcerans* NCTC 7910^T^ (99.93%), *C. ramonii* FRC0011^T^ (99.86%), *C. pseudotuberculosis* ATCC 19410^T^ (99.71%), and *C. silvaticum* KL0182^T^ (99.64%). Molecular identification by analysis of the *rpo*B gene fragment showed similarity with the species *C. ulcerans* NCTC 7910^T^ (100%), *C. ramonii* FRC0011^T^ (97.09%), and *C. silvaticum* CVUAS 4292^T^ (97.04%). The analysis of both genes obtained results above the proposed cutoff points suggested by Khamis and collaborators [17, 34], making it impossible to predict the species by gene similarity.

Khamis and collaborators [17] have already observed ambiguities for some *Corynebacterium* spp., such as *C. pseudodiphtheriticum*/*C. propinquum* and *C. aurimucosum*/*C. minutissimum*. It should be considered that at the time of this study, the genus *Corynebacterium* comprised approximately 70 species. Currently, the diversity of the genus has increased considerably, comprising a total of 168 valid species [35], with their phenotypic relationships very closely related. It should also be emphasized that many of these new species came from taxonomic reclassification studies, and thus, obtaining findings like those of Khamis and collaborators [17] with ambiguities when analyzing the 16S rRNA gene.

**Fig. 1:**
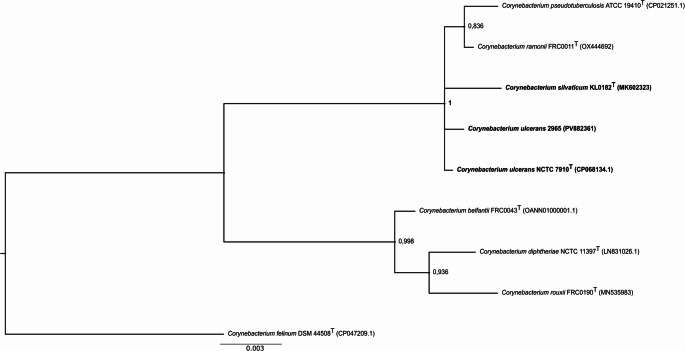
Bayesian phylogenetic tree of *Corynebacterium ulcerans* 2965 and related type strains based on 16S rRNA gene sequences. Inference was performed in MrBayes v3.2.7a under the HKY + I+F model (20,000,000 generations; 25% burn-in). Posterior probabilities are shown at the nodes. *C. felinum* DSM 44508 ^T^ served as outgroup. Scale bar: 0.003 substitutions/site

Regarding the phylogenetic study of the 16S rRNA (Fig. 1), we can observe a close phylogenetic relationship between the isolate *C. ulcerans* 2965 and the species *C. ulcerans* NCTC 7910^T^ and *C. silvaticum* KL0182^T^ since a polytomous clade comprising these three species was formed, which, although with high posterior support, does not evidence an interspecific differentiation. Furthermore, the relationship between the species *C. ramonii* FRC0011^T^ and *C. pseudotuberculosis* ATCC 19410^T^ cannot be disregarded since the terminal clade that includes these species is not phylogenetically resolved, mainly due to the moderate posterior support that was generated by the tree reconstruction (0.836). Our results are corroborated by the study by Khamis and collaborators [17], which concluded that the 16S rRNA gene does not have sufficient variation for *Corynebacterium* spp. to guarantee reliable results, despite presenting a high support value.

**Fig. 2:**
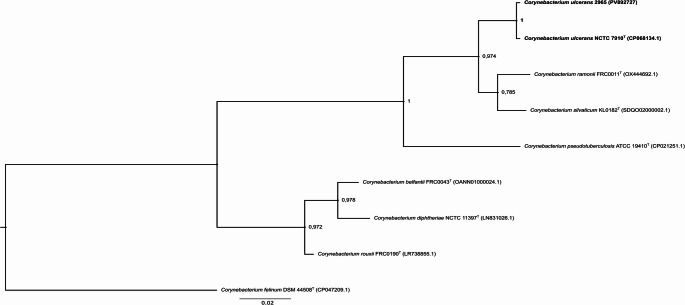
Bayesian phylogenetic tree of *Corynebacterium ulcerans* 2965 and related type strains based on partial *rpo*B gene sequences. Inference was performed in MrBayes v3.2.7a under the GTR + G+F model (20,000,000 generations; 25% burn-in). Posterior probabilities are shown at the nodes. *C. felinum* DSM 44508^T^ served as outgroup. Scale bar: 0.02 substitutions/site

On the other hand, the phylogenetic study based on the *rpo*B gene fragment allowed us to confirm the taxonomic identification of the isolate 2965 as *C. ulcerans*, resolving the ambiguity generated by the similarity and phylogeny of the 16S rRNA gene. As observed in Fig. 2, the formation of a terminal clade that includes the isolate *C. ulcerans* 2965 and *C. ulcerans* NCTC 7910^T^, with strong posterior support (1.00), reinforced by the innermost clade, also with strong posterior support (0.974), separates the clade from the species *C. ulcerans* and the clades of the related species *C. ramonii* FRC0011^T^ and *C. silvaticum* KL0182^T^. Although these last species were grouped in a terminal clade with a moderate posterior support (0.785), it is well resolved phylogenetically by the inner clade. Therefore, this result indicates that isolate *C. ulcerans* 2965 is phylogenetically related to this species, confirming its taxonomic identification.

Our results reinforce the importance of phylogenetic analysis for the delimitation of bacterial species, since approaches based solely on sequence similarity, such as BLAST, may be insufficient to differentiate phylogenetically close species [36, 37]. Furthermore, the use of the *rpo*B gene, as recommended by Khamis and collaborators [17] as an alternative or replacement in the identification of *Corynebacterium* spp., showed discriminatory power when analyzed phylogenetically. These findings reinforce that the use of more polymorphic genes, such as *rpo*B, provides greater phylogenetic resolution capacity for closely related species, overcoming the limitations observed in conserved genes such as 16S rRNA.

The mPCR technique was also applied to investigate the toxigenicity potential of the isolate, which was confirmed by amplification of a fragment compatible with fragment A of the DT of the species *C. diphtheriae* ATCC 27012, used as a positive control for this analysis. Once the methods based on PCR only allow the presumption of toxigenicity [31], a phenotypic test was carried out to confirm DT production.

The modified Elek test was negative for DT production by the *C. ulcerans* 2965 strain, evidenced by the absence of the immunoprecipitation line. The control strains *C. diphtheriae* ATCC 27010^T^ (*tox-*) and *C. diphtheriae* ATCC 27012 (*tox+*) showed reactions compatible with expectations, validating the test. Given this finding, *C. ulcerans* 2965 strain was considered a NTTB strain. Although NTTB isolates are not classified as toxigenic, they are epidemiologically relevant as potential reservoirs of the *tox* gene, since the ability to express the DT can be restored. It is recommended to maintain genomic and laboratory surveillance and adopt appropriate public health measures for contacts and animals involved [38].

Presently, the isolate was additionally submitted to an antimicrobial susceptibility test. It showed that the isolate was susceptible to cefotaxime, meropenem, erythromycin, tetracycline, linezolid, rifampicin, and trimethoprim-sulfamethoxazole. However, the isolate presented a susceptible, increasing exposure profile to benzylpenicillin and ciprofloxacin. No resistance was identified. Table 1 shows the result of the susceptibility profile with the respective sizes of inhibition halos.

**Table 1:** Susceptibility profile of *Corynebacterium ulcerans* 2965 isolate

Antimicrobial	BrCAST Standard			Inhibition halo diameter (mm)	Profile
	S ≥	I	*R*<		
Benzylpenicillin	50	12–49	12	25	I
Cefotaxime	50	15–49	15	26	I
Meropenem	24	-	24	38	S
Ciprofloxacin	50	24–49	24	35	I
Erythromycin	24	-	24	38	S
Tetracycline	24	-	24	30	S
Linezolid	25	-	25	36	S
Rifampicin	24	-	24	39	S
Trimethoprim-sulfamethoxazole	23	-	23	36	S

Legends: S, susceptible; I, susceptible, increased exposure; R, resistant.

*C. ulcerans* is frequently isolated as a colonizer or infectious agent from companion animals such as dogs, cats, and pigs. Cases of diphtheria due to *C. ulcerans* strains are not uncommon and have been associated with zoonotic transmission. In some European countries, the number of diphtheria cases due to this species has already surpassed those caused by *C. diphtheriae* [39, 40]. Unfortunately, we do not have information regarding the contact of the patient with pets or other animals.

In Brazil, only one case of infection caused by a *C. ulcerans* strain carrying the *tox* gene was found in the literature [41]. However, more robust analyses carried out later demonstrated that this strain did not harbor the gene [42]. Since then, other studies have reported the isolation of *C. ulcerans* strains from humans and other animals, but none have detected the *tox* gene [43–45]. Thus, the present work is the first to report the isolation of a *tox* + *C. ulcerans* strain from a diphtheria case in Brazil.

Although the isolate was characterized as susceptible to all tested antimicrobials and cannot produce the DT, the isolation of a *C. ulcerans* strain from a human diphtheria case serves as a warning to public health surveillance services in Brazil and reinforces the need to maintain vaccination against diphtheria at ideal levels to prevent outbreaks and epidemics.
